# Chemical Composition, Antioxidant, Anti-Tyrosinase, Anti-Cholinesterase and Cytotoxic Activities of Essential Oils of Six Algerian Plants

**DOI:** 10.3390/molecules25071710

**Published:** 2020-04-08

**Authors:** Kadour Cheraif, Boulanouar Bakchiche, Abdelaziz Gherib, Sanaa K. Bardaweel, Melek Çol Ayvaz, Guido Flamini, Roberta Ascrizzi, Mosad A. Ghareeb

**Affiliations:** 1 Laboratory of Process Engineering, Faculty of Technology, Amar Telidji University, Laghouat 03000, Algeria; cheraifkadour@yahoo.fr (K.C.); b.bakchiche@lagh-univ.dz (B.B.); a.gherib@lagh-univ.dz (A.G.); 2 Department of Pharmaceutical Sciences, School of Pharmacy, University of Jordan, Amman 11942, Jordan; s.bardaweel@ju.edu.jo; 3 Department of Chemistry, Faculty of Science and Arts, Ordu University, 52200 Ordu, Turkey; melekcol@hotmail.com; 4 Dipartimento di Farmacia, Università di Pisa, Via Boanno 6, 56126 Pisa, Italy; roberta.ascrizzi@gmail.com; 5 Medicinal Chemistry Department, Theodor Bilharz Research Institute, Kornaish El-Nile, Warrak El-Hadar, Imbaba (P.O. 30), Giza 12411, Egypt; m.ghareeb@tbri.gov.eg

**Keywords:** *Artemisia campestris*, *Artemisia herba-alba*, *Juniperus phoenicea*, *Juniperus oxycedrus*, *Mentha pulegium*, *Lavandula officinalis*, essential oils, GC/MS, antioxidant activity, acetylcholinesterase, butyrylcholinesterase, tyrosinase

## Abstract

In this study, the essential oils (EOs) of six Algerian plants (*Artemisia campestris* L., *Artemisia herba-alba* Asso, *Juniperus phoenicea* L., *Juniperus oxycedru*s L., *Mentha pulegium* L. and *Lavandula officinalis* Chaix) were obtained by hydrodistillation, and their compositions determined by GC-MS and GC-FID. The antioxidant activity of the EOS was evaluated via 2,2′-diphenyl-1-picrylhydrazyl (DPPH), ferric-reducing/antioxidant power (FRAP) and 2,2′-azino-bis(3-ethylbenzothiazoline-6-sulphonic acid (ABTS) assays. Moreover, their cytotoxic effect was evaluated—as well as their tyrosinase, acetyl- and butyryl-cholinesterase (AChE and BuChE) inhibitory activities. The chemical analyses detected 44, 45, 51, 53, 26 and 40 compounds in EOs of *A. campestris*, *A. herba-alba*, *J. phoenicea*, *J. oxycedrus*, *M. pulegium* and *L. officinalis*, respectively. *A. campestris* EO was mainly composed of β-pinene (20.7%), while *A. herba-alba* EO contained davanone D (49.5%) as the main component. α-Pinene (41.8%) was detected as the major constituent in both *J. phoenicea* (41.8%) and *J. oxycedrus* (37.8%) EOs. *M. pulegium* EO was characterized by pulegone as the most abundant (76.9%) compound, while linalool (35.8%) was detected as a major constituent in *L. officinalis* EO. The antioxidant power evaluation revealed IC50 values ranging from 2.61 to 91.25 mg/mL for DPPH scavenging activity, while the FRAP values ranged from 0.97–8.17 µmol Trolox equivalents (TX)/g sample. In the ABTS assay, the values ranged from 7.01 to 2.40 µmol TX/g sample. In the presence of 1 mg/mL of the samples, tyrosinase inhibition rates ranged from 11.35% to 39.65%, AChE inhibition rates ranged from 40.57% to 73.60% and BuChE inhibition rates ranged from 6.47% to 72.03%. A significant cytotoxic effect was found for *A. herba-alba* EO. The obtained results support some of the traditional uses of these species in food preservation and for protection against several diseases.

## 1. Introduction

Essential oils (EOs) are complex mixtures of volatile compounds biosynthesized by plants in response to environmental and ecological needs [1]. In traditional medicines, EOs have been used since ancient times in the treatment of various ailments and health disorders. They have been extensively investigated for their important biologic applications, such as antimicrobial [2], antioxidant [3], antiproliferative [4], antimalarial and trypanocidal [5] and antitumoral [6]. Among the most useful EO applications, their use as antioxidant agents is highly investigated, since the phenomenon known as oxidative stress is the root of several health problems, like inflammations, cancer, neurodegeneration and cardiovascular diseases. The over-production of free radicals, highly energetic molecules containing odd electrons, mostly represented by reactive oxygen species (ROS), is involved in this phenomenon; as naturally occurring antioxidant agents, EOs may attenuate this damage through their radical scavenging effect [7,8,9,10,11]. Among the neurodegenerative diseases sustained by inflammation, Alzheimer′s disease (AD) represents the predominant reason of dementia in old people. Memory and language impairment, cognitive dysfunction and behavioral disturbances are common symptoms of AD. Moreover, the reduction in acetylcholine (ACh) levels in the hippocampus and cortex of the brain is a common biochemical change detected in AD patients. Consequently, the starting point for the treatment of AD is the inhibition of acetylcholinesterase (AChE) and butyrylcholinesterase (BChE), both responsible for the hydrolysis of ACh in the cholinergic synapse [12,13,14,15,16,17].

In this framework, the study of widely distributed species rich in EOs as sources of naturally occurring bioactive agents is of great importance, in order to exploit their large bioavailability. In the present work, attention was focused on six species belonging to the Algerian flora: *Artemisia campestris* L., *Artemisia herba-alba* Asso, *Juniperus phoenicea* L., *Juniperus oxycedrus* L., *Mentha pulegium* L. and *Lavandula officinalis* Chaix. An overview of the popular names, traditional medicinal uses and main EO compounds of the Algerian species selected for the present study is reported in Table 1.

The genus *Juniperus* (Cupressaceae) comprises nearly 75 species, widely distributed in the Northern hemisphere, especially in Tunisia, Algeria and Morocco. Numerous studies reported the chemical composition and biologic activities of the EOs from different species of this genus [26,27,28,29]. *Juniperus oxycedrus* L. is shrub or small tree, native to the Mediterranean region; it has been used throughout history for several medical applications [29,30]. *Juniperus phoenicea* is an evergreen tree native to North Africa: traditionally, this plant was used for the treatment of hypoglycemia [31], diarrhea, rheumatism [32] and diabetes [32,33]. The genus *Artemisia* (Asteraceae) contains approximately 400 species, widely distributed in the Mediterranean region, Northern Africa, Western Asia, Southwestern Europe and Arabian Peninsula [34]. *Artemisia herba-alba* is a greenish-silver perennial herb belonging to the Asteraceae family, with many popular names: in Algeria it is known as the white wormwood, in Arabic as “Chih”, and in France as “Armoise blanche” [35,36,37]. In traditional medicine, the plant has been used to treat many ailments including colds, coughing, bronchitis, intestinal disturbances, diarrhea, neuralgias arterial hypertension and/or diabetes [38,39,40]. For *A. herba-alba* EO, numerous biologic and pharmacological properties are reported in the literature, such as antimicrobial, antioxidant, antidiabetic, antileishmanial, anthelmintic and antispasmodic [36,37,41,42,43,44,45]. *Artemisia campestris* L. is a perennial herb, usually known as field wormwood. The plant is widely spread in Asia, North America, Europe and North Africa [46]. For this species, several ethnopharmacological uses are reported, such as anti-diabetic, anti-inflammatory, antioxidant, antimicrobial and antipyretic [47,48]. *Lavandula*, of the Lamiaceae family, is an aromatic genus with about 39 species distributed worldwide, from the Mediterranean region, to tropical Africa and the South-East regions of India [49,50,51]. It was traditionally used as spasmolytic, carminative, stomachic and diuretic [50]. Leaf and flower parts of most *Lavandula* species are rich in essential oils [50,52,53]. *Lavandula officinalis* Chaix. is a multifunctional medicinal and aromatic plant native to Southern Europe and the Mediterranean region [54], widely used in both the pharmaceutical and fragrance industries. The genus *Mentha* belongs to the *Lamiaceae* family: it is broadly distributed worldwide [55], comprising about 19 species and 13 natural hybrids, basically perennial herbs, growing in Europe, Asia, Africa, Australia and North America [55,56]. In folk medicine, *Mentha* species have been used for treatment of various ailments including nausea, bronchitis, flatulence, anorexia, ulcerative colitis and liver complaints, due to their anti-inflammatory, carminative, antiemetic, diaphoretic, antispasmodic, analgesic, stimulant, emmenagogue and anticatarrhal activities [56,57,58,59,60].

Because of the diverse chemical and biologic profiles of the selected species, the present study aimed at investigating the chemical profile of the essential oils (EOs) from these six Algerian plants by GC-MS analysis. Finally, their antioxidant, anti-tyrosinase and cytotoxic activities were evaluated.

## 2. Results

### 2.1. Chemical Characterization of the Essential Oils

**Figure 1:**
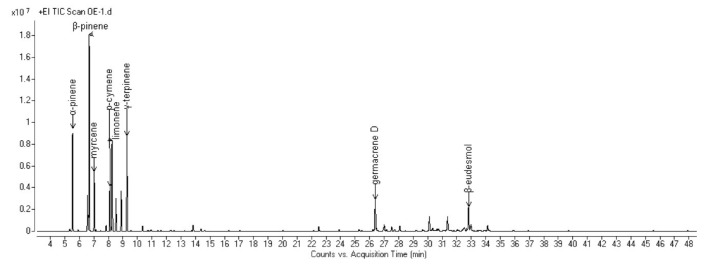
GC chromatogram of the EO hydrodistilled from the aerial parts of *A. campestris* L.

**Figure 2:**
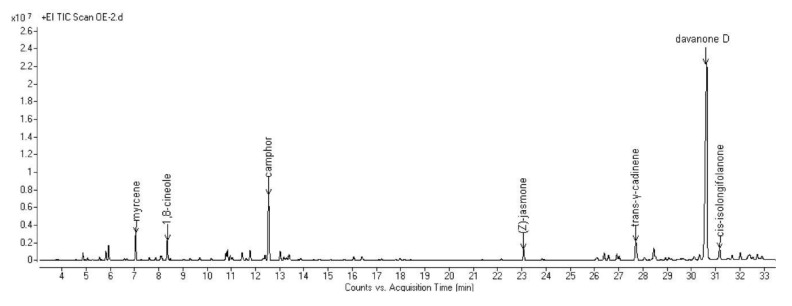
GC chromatogram of the EO hydrodistilled from the aerial parts of *A. herba-alba* Asso.

**Figure 3:**
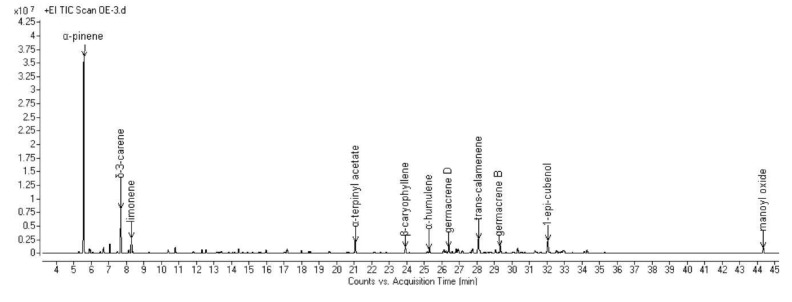
GC chromatogram of the EO hydrodistilled from the aerial parts of *J. phoenicea* L.

**Figure 4:**
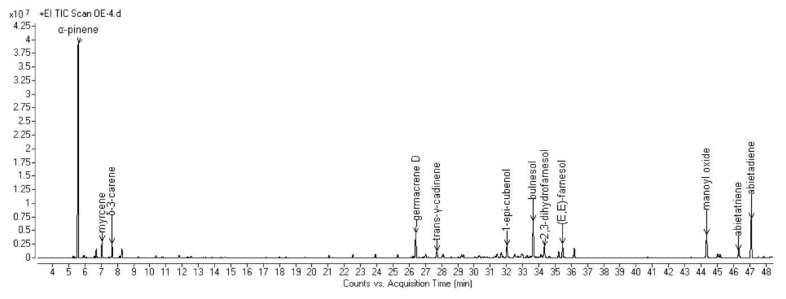
GC chromatogram of the EO hydrodistilled from the aerial parts of *J. oxycedrus* L.

**Figure 5:**
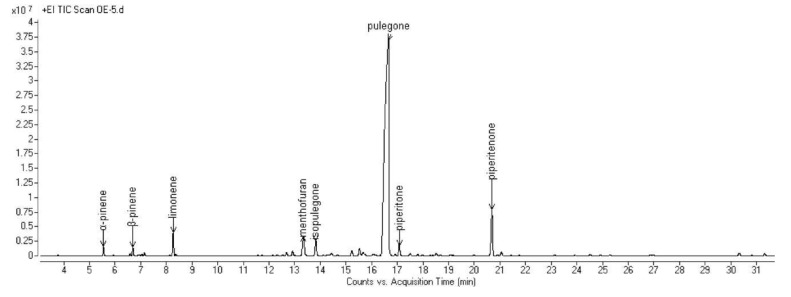
GC chromatogram of the EO hydrodistilled from the aerial parts of *M. pulegium* L.

**Figure 6:**
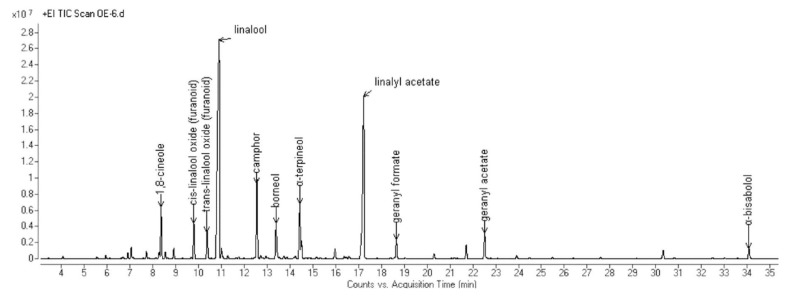
GC chromatogram of the EO hydrodistilled from the aerial parts of *L. officinalis* Chaix.

The complete compositions of the essential oils (EOs) hydrodistilled from the aerial parts of the six selected Algerian species are reported in Table 2. All the obtained chromatograms are reported in Figure 1, Figure 2, Figure 3, Figure 4, Figure 5 and Figure 6. For each species, the hydrodistillation was performed in triplicate: the average yields (*w*/*w*) of extraction were 0.52%, 0.54%, 0.4%, 0.19%, 0.91% and 2.80%, respectively for *Artemisia campestris*, *Artemisia herba-alba*, *Juniperus phoenicea*, *Juniperus oxycedrus*, *Mentha pulegium* and *Lavandula officinalis*.

Forty-four compounds were identified in *A. campestris* EO, representing 99.1% of the total composition. Monoterpene hydrocarbons were the most prominent chemical class of compounds, accounting for up to 75.7%: among them, β-pinene (20.7%), limonene (11.3%), γ-terpinene (11.0%), α-pinene (9.2%), myrcene (5.8%) and *p*-cymene (5.1%) were the most abundant. Sesquiterpenes followed, with similar relative abundances in their hydrocarbon and oxygenated forms (9.0% and 10.0%, respectively), exhibiting germacrene D (4.9%) and β-eudesmol (3.9%) as the most represented compounds of the two classes, respectively.

Forty-five compounds were identified in *A. herba-alba* EO, adding up to 92.2% of the total composition, constituted for more than 70% by oxygenated terpenes. *A. herba-alba* EO was composed for more than 50% by oxygenated sesquiterpenes, among which the most abundant was davanone D (49.5%). Among the monoterpenoids, camphor (10.0%) was the most represented.

In *J. phoenicea* EO, fifty-one compounds were identified, representing 97.5% of the composition. Terpene hydrocarbons were the most abundant compounds: monoterpene hydrocarbons, in particular, accounted for almost 60% of the total. Among them, the most abundant was α-pinene, which represented over 40% of the composition, followed by δ-3-carene (8.4%). For the sesquiterpenes, *trans*-calamenene showed the highest relative abundance (4.2%) within the hydrocarbons, while 1-*epi*-cubenol was the main (3.7%) oxygenated one.

Fifty-three compounds were detected in *J. oxycedrus* EO, representing 95.8% of the composition. Monoterpene hydrocarbons were the main chemical class (46.3%), among which α-pinene accounted for over 35%, thus representing the most abundant compound in the composition. Oxygenated sesquiterpenes followed (21.4%), with bulnesol as the most represented (7.2%). Diterpenes were also relevantly represented: abietadiene showed a relative abundance over 8%, while manoyl oxide was up to 5%.

*M. pulegium* EO contained twenty-six volatile constituents, representing 96.8% of the total. Its composition was dominated (over 90%) by oxygenated monoterpenes: the plant, indeed, exhibited a pulegone chemotype [61], as this compound was the most abundant (76.9%) in the EO, followed by piperitenone (6.05%).

Forty compounds were identified in *L. officinalis* EO, representing 98.5% of the total. Almost 90% of its composition was constituted by oxygenated monoterpenes, of which linalool and linalyl acetate were the most abundant, as they accounted for up to 35.8% and 21.0%, respectively.

### 2.2. Antioxidant Activity

The EOs antioxidant activity is reported in Table 3: it was evaluated as 2,2′-diphenyl-1-picrylhydrazyl (DPPH) free radical scavenging activity, ferric-reducing/antioxidant power (FRAP) and 2,2′-azino-bis(3-ethylbenzothiazoline-6-sulphonic acid (ABTS) radical scavenging activity assays.

In all the antioxidant assays, all the EOs exhibited a lower antioxidant power compared to ascorbic acid, used as a positive control. *A. herba-alba* EO had the highest antioxidant activity in both the DPPH and the FRAP assays. A high Pearson′s correlation coefficient (0.82) was found between DPPH and FRAP values. However, such high correlation values could not be obtained in the case of ABTS test results, which evidenced *A. campestris* as the best-performing antioxidant EO. *J. oxycedrus* EO scored the lowest antioxidant power in both the DPPH and FRAP assays.

### 2.3. Acetylcholinesterase (AChE), Butyrylcholinesterase (BuChE) and Tyrosinase Inhibitory Activities

The EOs inhibitory activity on the three selected enzymes is reported in Table 4. All the tested EOs exhibited inhibiting activities lower than the control standard in all the enzymatic assays. In the AChE inhibition assay, *L. officinalis* EO was detected as the best-performing, followed by *M. pulegium* EO. *M. pulegium* EO, instead, was the most active sample in the BuChE inhibition assay, with a rate close to the positive control. In both the AChE and BuChE inhibition assays, *A. campestris* and *J. phoenicea* EOs exhibited the lowest inhibitory activity among the six EOs. With the exception of *J. phoenicea*, all the EOs showed a similar degree (around 30%) of tyrosinase inhibition potential.

### 2.4. Cytotoxic Activity

The EOs anticancer activities were evaluated on three human cancer cell lines, including two human breast adenocarcinoma (MCF-7 and T-47D) and one human colon cancer (Caco-2) cell lines. The LD_50_ values, labeled as the concentration at which 50% of cell growth is inhibited, are presented in Table 5. Results indicated that the LD_50_ values were in the range of 0.016–0.99 mg/mL, with the MCF-7 cancer cell line as the most responsive to treatment. *A. herba-alba* EO demonstrated the most potent activity among the examined EOs.

## 3. Discussion

*A. campestris* EO exhibited a predominance of oxygenated monoterpenes, differing from what was reported for an accession from South-East Morocco, in whose EO composition oxygenated sesquiterpenes were detected as the most abundant class of compounds, with spathulenol and β-eudesmol as the most represented [62]. For β-pinene, the most abundant compound in this EO, a broad range of biologic activities are reported, such as antimicrobial [63], anticonvulsant [64], gastroprotective [65], antioxidant [66] and neuroprotective [64]. Moreover, its combination with paclitaxel showed a synergistic effect on non-small-cell lung cancer (NSCLC), thus confirming the importance of the evaluation of the combined use of EOs rich in compounds with reported in vitro anti-proliferative activity in cancer chemotherapy [67]. Compositional differences with published literature also emerged in the EO of *A. herba-alba*; while the accession of the present study was mainly composed of oxygenated sesquiterpenes, specimens from Algeria [34,37] and Morocco [68] were reported as predominantly composed of oxygenated monoterpenes. *J. phoenicea*, instead, showed the same α-pinene chemotype as reported for five specimens from Eastern Algeria [28] and one from the Boulmane region of Morocco [69]. The other *Juniperus* species studied in the present work presented a pulegone chemotype, as previously published analyses performed on accessions from Tunisia [29], Central Italy [70], Bulgaria and Serbia [71]. A predominance of sesquiterpenes, followed by diterpenes, was, instead, reported for a Tunisian accession of *J. oxycedrus* [70]. A pulegone chemotype was also found, in this study, for *M. pulegium*, in accordance with Sbayou et al. (2016) and Politeo et al. (2018), who analyzed the composition of two EOs of Iranian and Bosnia-Herzegovina specimens, respectively [68,72]. This oxygenated monoterpene exhibited the ability to inhibit the chemical and thermal nociceptive central perception in vivo [73]. An accession from North-West Iran, instead, was reported as mainly rich in menthone, thus exhibiting the second possible chemotype for this species [54]. The predominance of the oxygenated monoterpenes class found for *L. officinalis* EO of the present study was also reported by Marín et al. (2016), who analyzed an EO a Spanish accession [74]; moreover, linalool was reported as the main constituent of the flower EO of an Iranian specimen [75], while its acetic ester was predominant in a Brazilian accession [76]. Instead, 1,8-cineole and borneol were reported as the most abundant components of the EOs extracted from other two Iranian specimens [50,77]. Algerian *L. officinalis*, with its linalool-rich EO, may be considered a source of this oxygenated monoterpene, for which a wide range of bioactivities are reported, such as anti-inflammatory, anticancer, anti-hyperlipidemic, antimicrobial, antinociceptive, analgesic, anxiolytic, antidepressant and neuroprotective [78].

The highest antioxidant activity of *A. herba-alba* in the DPPH and FRAP assays may be due to its relevant (10.0%) relative abundance of camphor, reported as favorable in terms of antioxidant power in an *Artemisia judaica* EO [79]. Its higher antioxidant activity in the ABTS assay may be due to the predominance of oxygenated terpenes, reported as more effective in neutralizing free radicals, quenching singlet and triplet oxygen, decomposing peroxides and chelating transition metals [80]. The lowest antioxidant power shown by *J. oxycedrus* EO, instead, may be due to its high relative content (over 35%) of α-pinene, for which a negative relationship with the antioxidant activity has been reported [81]. The contribution of single EO compounds to their antioxidant activity is still object of many debates. Mimica-Dukic et al. (2003) and Yadegarinia et al. (2006) reported that oxygenated monoterpenes act as radical scavenging compounds [82,83]. Ruberto and Baratta (2000), instead, indicated monoterpene hydrocarbons as responsible compounds for the antioxidant activity [84]. The importance of compounds in lower relative abundances, though, must be taken into account, considering the overall activity of the EOs with a more holistic approach, in which the phytocomplex acts as the result of the contribution of all the single components, which, ultimately, are effective in synergy [85]. Moreover, there are reports of quantitatively minor components exerting the antioxidant activity over the most abundant ones [86].

The inhibitory activity of the selected EOs on acetylcholinesterase (AChE) and butyrylcholinesterase (BuChE) cholinesterase (ChE) were studied since these enzymes have a fundamental role in the nervous system, as they are responsible for the hydrolysis of ACh [87]. *L. officinalis* and *M. pulegium* EOs, both rich in oxygenated terpenes, showed the highest inhibitory potency on AChE. This is in accordance with Benabdallah et al. (2018), who reported this chemical class of compounds and especially 1,8-cineole, whose highest relative abundance in the samples of the present study was found in *L. officinalis* EO, as the reason for the AChE inhibition activity [88]. Miyazawa et al. (1998) and Öztürk (2012) also attributed the AchE potency of *Mentha* spp. EOs to their oxygenated monoterpene fraction [89,90]. Monoterpenoids, indeed, may act as competitive or non-competitive inhibitors of the cholinesterase enzymes, with which they may be able to interact, due to their lipophilicity, on their hydrophobic sites [91]. *M. pulegium* EO was also found as the most active inhibitor of BuChE; since this enzyme increases in patients′ brains as the Alzheimer′s disease (AD) progresses, this result on the BuChE inhibiting activity is important, as this cholinesterase may be a better target for the AD therapy compared to AChE, especially in the late stages of the disorder [92]. The lowest inhibitor activity towards these enzymes was found for *A. campestris* and *J. phoenicea* EOs; their compositions showed a lower presence of oxygenated monoterpenes in favor of a higher relative content of their hydrocarbon counterparts. However, exactly like the antioxidant power of EOs, their inhibiting activity on cholinesterase enzymes is most probably due to their complete composition, in which the phytocomplex compounds act in synergy [89,93].

The EOs of the selected species were also tested for their inhibitory activity on tyrosinase: as this enzyme is involved in the first steps of melanin biosynthesis inside the melanocytes [94], its inhibition may be used to modulate skin pigmentation in hyperpigmentation disorders. Moreover, alterations in this biosynthetic step are involved in the development of some histopathological features of malignant metastatic melanoma, thus its inhibition represents a viable target to treat skin cancer [95]. EOs ability to inhibit this enzyme has been reported as competitive, non-competitive or mixed, based on their chemical composition [96]. Due to the complexity of the EO compositions, this activity is most probably attributable to a synergistic interaction of their compounds with the enzyme, rather than to a single component [97,98]. Contrary to published reports [96,99], however, the anti-tyrosinase activity does not seem correlated to the oxygenated monoterpenes fraction in the EOs composition: the two best performing ones in our assay were, indeed, the EOs with the lowest relative abundances of this chemical class of compounds.

The MCF-7 (human breast adenocarcinoma) cancer cell line was the most responsive to the EO treatments. *A. herba-alba* EO showed the highest cytotoxic potency. This may be explained by its high davanone D content, whose relative content added up to almost 50% of the EO. For this compound and its derivatives, anti-proliferative and pro-apoptotic effects are reported for several cancer cell lines, included MCF-7 [100]. Moreover, its unique chemical composition is rich in known naturally occurring anticancer chemical ingredients such as α-thujone (28.1%), camphor (22.8%) and 1,8-cineole (8.2%) [101]. There are very few published studies in the literature that reported the anticancer activity of *A. herba-alba* EO. Our results demonstrated that the essential oil had significant anticancer activity against the examined breast and colon cancer cells. The observed activity started at very low EO concentrations (lower than 5 µg/mL), while more than 80% of the cells were in the lysis phase at high concentration (300 µg/mL), suggesting a great potential for a naturally occurring chemotherapeutic or chemo-preventive agent.

## 4. Materials and Methods

### 4.1. Plant Material

For all the species, 1 kg of aerial parts was individually collected at fruiting stage during May–July 2018 in Laghouat, Algeria (Latitude: 33°47′59″, Longitude: 2°51′54″, Altitude: 764 m). The identification and authentication of the plants were carried out by the botanist Dr. Mohamed Kouidri (Department of Agronomy, Faculty of Sciences, University of Laghouat, Laghouat, Algeria) and the voucher specimens were deposited at the Laboratory of Process Engineering, University of Laghouat, Algeria with the numbers LGP Ac/07/18, LGP Ah/07/18, LGP Jp/05/18, LGP Jo/05/18, LGP Mp/06/18 and LGP Lo/06/18, respectively for *Artemisia campestris*, *Artemisia herba-alba*, *Juniperus phoenicea*, *Juniperus oxycedrus*, *Mentha pulegium* and *Lavandula officinalis*.

### 4.2. Essential Oil Extractions

For each species, 100 g of air-dried aerial parts of the collected plants was hydrodistilled for 3 h using a Clevenger-type apparatus. The obtained essential oils (EOs) were dried over anhydrous sodium sulphate and, after filtration, stored at 4 °C until analysis.

### 4.3. Gas Chromatography–Mass Spectrometry Analyses and Peaks Identification

The hydrodistilled essential oils were diluted to 0.5% in HPLC-grade *n*-hexane and then injected into a GC—MS apparatus. Gas chromatography–electron impact mass spectrometry (GC–EIMS) analyses were performed with an Agilent 7890B gas chromatograph (Agilent Technologies Inc., Santa Clara, CA, USA) equipped with an Agilent DB-5MS (Agilent Technologies Inc., Santa Clara, CA, USA) capillary column (30 m × 0.25 mm; coating thickness 0.25 μm) and an Agilent 5977B single quadrupole mass detector (Agilent Technologies Inc., Santa Clara, CA, USA). The analytical conditions were as reported in Zardi-Bergaoui et al. (2018) [102]: injector and transfer line temperatures 220 and 240 °C, respectively; oven temperature programmed from 60 to 240 °C at 3 °C/min; carrier gas helium at 1 mL/min; injection of 1 μL (0.5% HPLC grade *n*-hexane solution); split ratio 1:25. The acquisition parameters were as follows: full scan; scan range: 30–300 *m*/*z*; scan time: 1.0 s.

The GC analyses were accomplished with a HP-5890 Series II instrument (Agilent Technologies Inc., Santa Clara, CA, USA) equipped with a HP-5 (Agilent Technologies Inc., Santa Clara, CA, USA) capillary columns (30 m × 0.25 mm, 0.25-μm film thickness), set to the following conditions: temperature program of 60 °C for 10 min, followed by an increase of 3 °C /min to 220 °C; injector and detector temperatures at 250 °C; carrier gas helium (1 mL/min); detector FID; split ratio 1:30). The relative proportions of the individual constituents, expressed as percentages, were obtained by FID peak-area normalization.

As reported in Mosbah et al. (2018) [103], the identification of the constituents was based on the comparison of the retention times with those of authentic samples, comparing their linear retention indices relative to the series of *n*-hydrocarbons, and on computer matching against commercial (NIST 2014 and ADAMS 2007) and home-made library mass spectra built up from pure substances and components of commercial essential oils of known composition and MS literature data [104,105,106].

### 4.4. Antioxidant Assays

#### 4.4.1. DPPH Free Radical Scavenging Activity

The DPPH free radical scavenging activity of the hydrodistilled essential oils was evaluated by bleaching of the purple-colored methanol solution of 2,2′-diphenyl-1-picrylhydrazyl (DPPH) at 517 nm after the addition of extract at different concentrations as antioxidant agents to the DPPH solution. The inhibition concentration values obtained for each concentration was calculated using following equation:

Inhibition concentration (%) = [(A_blank_ − A_sample_)/A_blank_] × 100

IC_50_ values (extract concentrations providing 50% inhibition) were also calculated [107].

#### 4.4.2. Ferric-Reducing/Antioxidant Power

The FRAP assay was performed following the method based on the principle of reducing the Fe (III)-TPTZ complex in the presence of antioxidants to form blue Fe (II)-TPTZ complex and the subsequent measurement of the maximum absorbance at 595 nm [108]. For this purpose, appropriate amounts of the essential oil or Trolox standard were combined with the FRAP reagent (300 mM acetate buffer (pH 3.6), 10 mM 2,4,6-tripyridyl-s-triazine (TPTZ) solution prepared in 40-mM HCl and 20-mM FeCl_3_·6H_2_O in a 10:1:1 ratio just before use and heated to 37 °C). The mixtures were incubated at 37 °C for 30 min, then the resulting absorbances were measured at 593 nm. FRAP values for samples were calculated as Trolox equivalents (µmol TX/g sample).

#### 4.4.3. ABTS Radical Scavenging Activity

The ABTS radical scavenging activity was determined according to the method described by Re et al. (1999) [109] with some modifications. The ABTS solution was prepared by dissolving ABTS (2,2′-azino-bis(3-ethylbenzothiazoline-6-sulphonic acid) in water to a 7-mM concentration. ABTS radical cation (ABTS•+) was also produced by reacting ABTS solution with 2.45 mM potassium persulfate (final concentration) and allowing the mixture to stand in the dark at room temperature for 12–16 h before use. Then, the absorbance of the final ABTS radical solution was adjusted to 0.7 at 734 nm. The essential oil samples or Trolox standards at appropriate concentrations were combined with the stabilized radical solution and incubated at 30 °C. After 30 min, the absorbances were spectrophotometrically measured at 734 nm. Results were expressed as Trolox equivalents (µmol TX/g sample). Ascorbic acid was used as positive control.

### 4.5. Inhibitory Activity on AChE and BuChE Enzymes

Acetylcholinesterase (AChE) and butyrylcholinesterase (BuChE) inhibitory activities were measured by following the method developed by Ellman et al. (1961) [110]. Electric eel AChE and BuChE from equine serum were used as enzymes, while acetylthiocholine iodide and butyrylthiocholine chloride were used as substrates. The reaction mixture was first prepared to contain 0.2 M 5,5′-dithio-bis(2-nitrobenzoic) acid (DTNB) and 0.2-M enzyme solution in the presence of the sample tested as an inhibitor or of the standard inhibitor and incubated for 15 min at 25 °C. The reaction was then initiated with the addition of 0.2 M of each substrate. The hydrolysis of the substrates was monitored by the formation of the yellow 5-thio-2-nitrobenzoate anion as a result of the reaction of DTNB with thiocholines, catalyzed by enzymes at 412 nm (Abs_sample_). AChE/BuChE inhibition percentage was determined by comparison of the reaction rates of the samples relative to a blank sample (methanol as extraction solvent in phosphate buffer, pH 8) using the following equation:

Inhibition ratio (%) = [(Abs_blank_ − Abs_sample_)/Abs_blank_] × 100

Galantamine, an alkaloid-type anticholinesterase, was used as positive control.

### 4.6. Anti-Tyrosinase Activity

To evaluate the anti-tyrosinase activity of the essential oils, mushroom tyrosinase (0.5 mg/mL) was first incubated with each extract in phosphate buffer (50 mM, pH 6.8) for 20 min at room temperature. Following incubation, 0.5 mM of L-DOPA as substrate was added to this mixture and the change in absorbance at 475 nm as an indication of the enzymatic reaction due to formation of DOPA chrome was monitored. The percent of inhibition of tyrosinase reaction was calculated by using the same equation used for AChE/BuChE inhibition activity. Kojic acid was used as reference standard inhibitor for comparison [111].

### 4.7. Cytotoxic Activity

All cell lines (MCF7, T47D and Caco-2) were purchased from the American Type Culture Collection (Rockville, MD, USA). Cells were cultured in DMEM medium (Dulbecco′s Modified Eagle′s Medium), supplemented with 10% fetal bovine serum, 100 U/mL of penicillin, 100 µg/mL of streptomycin, at 37 °C with 5% of CO_2_. The count of viable cells was determined using the Trypan blue method, as previously described [112]. The cytotoxic effects of the examined oils were evaluated using the MTT assay (3-[4, 5-dimethylthiazole-2-yl]-2,5-diphenyl-tetrazolium bromide) (Sigma-Aldrich, St. Louis, MO, USA), as previously described [112]. The essential oils were tested for their cytotoxic activity in the concentration range of 0.001–10 mg/mL. Doxorubicin was employed as a positive control; the preparation and the treatment were performed in the same experimental conditions for the control and the test samples. Prism 8 software (GraphPad Software, San Diego, CA, USA) was utilized for data analysis to calculate inhibition percentages and the results were expressed as LD_50_ value, defined as the concentration that resulted in 50% growth inhibition of the cancer cell culture.

### 4.8. Statistical Analysis

All analyses were performed with the JMP^®^ Pro 13.2.1 (SAS Institute Inc., Cary, NC, USA) software. All the assays were performed in triplicate. The results are expressed as mean ± standard deviation (SD). The statistical significance of data in Table 3, Table 4 and Table 5 was evaluated using Tukey′s honest significance test (HSD), with α = 0.05.

## 5. Conclusions

Among the EOs studied in the present work, of all extracted from plants belonging to the Algerian flora, the highest antioxidant activity was evidenced for A*. herba-alba* and *M. pulegium* in both the DPPH and FRAP assays, while *A. campestris* showed the highest antioxidant power in the ABTS test.

*M. pulegium* EO also exhibited the strongest inhibiting power in the BuChE inhibition assay, while the best inhibitory effect on the AChE enzyme was evidenced for *L. officinalis* EO. The highest tyrosinase inhibition rates were found for *J. oxycedrus* and *A. campestris.* Finally, *A. herba-alba* EO exhibited remarkable cytotoxic effects on MCF-7, T47D and Caco-2 cancer cell lines.

These species should, thus, be re-evaluated as sources of value-added products such as EOs, whose numerous possible applications are based on their whole compositions, rather than on a single purified compound, isolated from the complete EO. Their application may range from food preservation—given their antioxidant power—to possible candidates to be added in the therapy of Alzheimer′s disease, as well as in cancer treatment. Further studies are needed to assess their efficacy in vivo. Moreover, close attention must be paid to the starting plant material, as many factors contribute to the overall chemical composition of the EOs: to ensure the best possible standardization of their chemical composition, indeed, the variations in the involved factors (genetic profile, geographical provenience, harvesting time, etc.) should be minimized.

## Figures and Tables

**Table 1:** Bibliographical overview of the popular names, traditional medicinal uses and main essential oils (EO) compounds of the Algerian species selected for the present study.

Family/Species	Local Name	Medicinal Uses in Algeria	Major Compounds of the Selected Species	References
**Asteraceae**				
*A. campestris*	Dgouft	Antidiabetic, antihypertensive	β-pinene (25%), sabinene (17%), α-pinene (9.9%), limonene (6.6%)	[18,19]
*A. herba-alba*	Chih	Antidiabetic, antispasmodic, carminative, anti-eczema	α-thujone (28.1%), camphor (22.8%), 1,8-cineole (8.2%)	[19,20,21]
**Cupressaceae**				
*J. phoenicea*	Aar-aar	Antidiarrheal, anti-rheumatic, antidiabetic, diuretic	α-pinene (75.8%), δ-3-carene (3.4%)	[18,22]
*J. oxycedrus*	Taga	Anti-inflammatory, anti-eye infections	*trans*-pinocarveol (7%), *cis*-verbenol (6.3%), manoyl oxide (6%)	[19,23]
**Lamiaceae**				
*M.* *pulegium*	Feliou	Antihypertensive,antispasmodic	pulegone (70.66%), *neo*-menthol (11.21%), menthone (2.63%)	[19,20,24]
*L. officinalis*	Khozama	antispasmodic, anti-influenza, treatments of abdominal pain	linalyl acetate (32.98), linalool (28.92%), lavandulyl acetate (4.52)	[20,25]

**Table 2:** Complete compositions of the essential oils hydrodistilled from the aerial parts of the six Algerian species selected in the present work.

Compounds	l.r.i. ^a^	Relative Abundance (%)					
		*Artemisia campestris*	*Artemisia herba-alba*	*Juniperus phoenicea*	*Juniperus oxycedrus*	*Mentha pulegium*	*Lavandula officinalis*
1-hexanol *	871	- ^b^	0.7	-	-	-	0.1
santolina triene	911	-	0.2	-	-	-	-
2,5-diethenyl-2-methyltetrahydrofuran	913	-	0.3	-	-	-	-
tricyclene	928	-	-	0.2	0.2	-	-
α-thujene	933	0.2	-	-	0.1	-	-
α-pinene *	941	9.2	0.3	41.8	37.8	0.7	0.1
α-fenchene	954	-	-	0.7	0.1	-	-
camphene *	955	0.1	1.7	0.5	0.3	-	0.2
3-methylcyclohexanone *	956	-	-	-	-	0.1	-
thuja-2,4(10)-diene	959	-	-	0.2	0.1	-	-
sabinene *	977	3.6	0.1	-	0.2	0.2	-
β-pinene *	982	20.7	0.2	1.0	1.1	0.8	0.1
3-octanone *	988	-	-	-	-	0.1	0.4
myrcene *	993	5.8	3.0	1.6	2.1	0.1	0.8
3-octanol *	994	-	-	-	-	0.2	0.2
α-phellandrene *	1006	-	-	0.3	0.1	-	-
1-hexyl acetate *	1010	-	-	-	-	-	0.5
δ-3-carene *	1011	-	-	8.4	1.9	-	-
α-terpinene *	1020	0.6	0.3	-	-	-	-
*p*-cymene *	1028	5.1	0.5	0.6	0.2	0.1	-
limonene *	1032	11.3	-	3.3	1.6	2.1	-
1,8-cineole *	1034	-	2.6	0.3	-	0.2	4.0
santolina alcohol	1039	-	0.2	-	-	-	-
(*Z*)-β-ocimene	1042	3.3	-	-	-	-	0.5
(*E*)-β-ocimene	1052	4.2	-	-	-	-	0.7
γ-terpinene *	1063	11.0	0.2	0.2	0.1	-	-
1-octanol *	1071	-	-	-	-	-	0.1
*cis*-linalool oxide (furanoid)	1076	-	-	-	-	-	2.9
artemisia alcohol	1084	-	0.2	-	-	-	-
terpinolene *	1090	0.6	-	0.8	0.4	-	-
*trans*-linalool oxide (furanoid)	1091	-	-	-	-	-	2.4
linalool *	1101	-	0.9	1.2	0.2	-	35.8
nonanal *	1102	0.1	-	-	-	-	-
α-thujone *	1104	-	0.3	-	-	-	-
1-octen-3-yl acetate *	1110	-	-	-	-	-	0.2
*cis*-linalool oxide (pyranoid)	1117	-	1.1	-	-	-	-
*cis*-*p*-menth-2-en-1-ol	1123	-	0.2	-	-	-	-
3-octyl acetate *	1124	-	-	-	-	-	0.1
chrysanthenone	1126	-	1.4	-	-	-	-
α-campholenal	1127	-	-	0.3	0.4	-	-
*trans*-pinocarveol	1141	0.1	0.3	0.7	0.1	-	-
*cis*-verbenol *	1142	-	-	-	0.1	-	-
*trans*-verbenol	1143	-	-	-	0.3	-	-
camphor *	1144	-	10.0	0.8	-	-	7.2
hexyl isobutyrate *	1151	-	-	-	-	-	0.2
menthone *	1154	-	-	-	-	0.4	-
nerol oxide	1155	-	-	-	-	-	0.2
β-pinene oxide	1157	-	1.2	-	-	-	-
*trans*-pinocamphone	1162	-	-	0.3	-	-	-
pinocarvone	1164	-	0.3	-	-	-	-
menthofuran *	1165	-	-	-	-	3.3	-
borneol *	1168	-	1.0	-	-	-	3.6
*cis*-linalool oxide (pyranoid)	1173	-	-	-	-	-	0.2
*iso*pulegone	1174	-	-	-	-	1.9	-
*trans*-linalool oxide (pyranoid)	1177	-	-	-	-	-	0.2
4-terpineol *	1179	0.7	0.3	-	-	-	0.1
cryptone	1185	-	-	-	-	-	0.2
α-terpineol *	1191	0.3	-	1.0	0.1	0.3	5.2
hexyl butyrate *	1192	-	-	-	-	-	1.5
myrtenol *	1193	0.1	0.2	-	0.1	-	-
verbenone *	1205	-	-	-	-	0.6	-
8,9-dehydrothymol	1222	-	-	-	-	0.5	-
citronellol *	1229	-	-	0.7	-	-	-
nerol *	1230	-	-	-	-	-	1.0
*nor*davanone	1231	-	0.5	-	-	-	-
hexyl 2-methylbutyrate *	1236	-	-	-	-	-	0.2
(*Z*)-3-hexenyl isovalerate	1239	0.1	-	-	-	-	-
pulegone *	1240	-	-	-	-	76.9	-
cumin aldehyde *	1241	-	-	-	-	-	0.2
piperitone *	1254	-	-	-	-	1.3	-
lepalone	1258	-	0.2	-	-	-	-
linalyl acetate *	1259	-	-	0.9	-	-	21.0
*iso*piperitone	1271	-	-	-	-	0.1	-
lepalol	1279	-	0.3	-	-	-	-
bornyl acetate *	1286	-	0.1	0.4	0.1	-	-
*trans*-linalool oxyde acetate (pyranoid)	1287	-	-	0.2	-	-	-
geranyl formate *	1297	-	-	-	-	-	1.8
carvacrol *	1298	-	-	-	-	0.1	-
(*E*,*E*)-2,4-decadien-1-ol	1311	-	-	0.5	-	-	-
hexyl tiglate *	1332	-	-	-	-	-	0.4
piperitenone	1342	-	-	-	-	6.0	-
α-terpinyl acetate *	1352	-	-	2.7	0.4	-	-
eugenol *	1358	-	0.2	-	-	-	-
neryl acetate *	1366	-	-	-	-	-	1.3
α-copaene *	1377	0.1	0.2	0.3	-	-	-
geranyl acetate *	1385	0.6	-	-	-	-	2.4
β-bourbonene	1386	-	-	0.2	0.5	-	-
β-elemene *	1392	-	-	0.2	-	-	-
(*Z*)-jasmone *	1395	-	1.6	-	-	-	-
*cis*,*cis*-nepetalactone	1397	-	-	-	-	0.1	-
β-caryophyllene *	1419	0.2	0.2	1.7	0.6	0.1	0.3
α-humulene *	1455	0.2	-	1.2	0.5	0.1	-
(*E*)-β-farnesene *	1459	0.1	-	-	-	-	0.1
*trans*-cadina-1(6),4-diene	1475	-	-	1.0	-	-	-
γ-muurolene	1478	0.2	-	0.5	0.2	-	-
germacrene D	1482	4.9	1.2	1.6	4.8	-	-
*ar*-curcumene	1483	0.3	-	-	-	-	-
β-selinene	1487	-	-	0.3	-	-	-
bicyclosesquiphellandrene	1489	-	-	1.1	-	-	-
davana ether	1491	-	1.0	-	-	-	-
valencene *	1492	-	-	1.1	-	-	-
viridiflorene	1493	-	0.6	-	-	-	-
bicyclogermacrene	1496	1.2	-	-	-	-	-
2-tridecanone *	1497	-	-	-	0.7	-	-
α-muurolene	1499	0.1	-	0.7	0.1	-	-
(*E*,*E*)-α-farnesene	1508	0.6	-	-	-	-	-
*trans*-γ-cadinene	1514	0.3	3.9	-	1.5	-	-
cubebol	1515	-	-	1.2	-	-	-
*trans*-calamenene	1523	-	-	4.2	0.7	-	-
δ-cadinene	1524	0.8	0.5	0.4	0.1	-	-
artedouglasia oxide A	1535	-	0.5	-	-	-	-
α-calacorene	1543	-	-	0.4	-	-	-
laciniatafuranone E	1544	-	0.5	-	-	-	-
elemol	1550	-	-	0.9	-	-	-
germacrene B	1557	-	-	1.9	0.5	-	-
(*E*)-nerolidol *	1564	0.2	-	-	-	-	-
spathulenol	1576	2.1	0.6	-	-	-	-
caryophyllene oxide *	1581	-	0.8	1.3	0.6	0.3	0.9
globulol *	1584	0.4	-	-	-	-	-
davanone D	1587	-	49.5	-	-	-	-
viridiflorol *	1591	0.3	-	-	-	-	-
*cis*-*iso*longifolanone	1605	-	2.1	-	-	-	-
geranyl isovalerate *	1606	2.3	-	-	-	-	-
humulene epoxide II	1607	-	-	0.7	0.3	0.2	-
β-atlantol	1608	-	-	-	0.8	-	-
humulane-1,6-dien-3-ol	1615	-	-	-	0.2	-	-
1-*epi*-cubenol	1629	-	-	3.7	2.6	-	-
γ-eudesmol	1631	0.3	-	-	-	-	-
*iso*spathulenol	1640	0.3	-	-	-	-	-
T-cadinol	1641	0.9	-	0.8	0.8	-	0.1
agarospirol	1645	0.4	-	-	0.3	-	-
β-eudesmol	1650	3.9	-	-	-	-	-
α-eudesmol	1651	-	-	0.6	-	-	-
α-cadinol	1652	1.2	-	0.3	0.5	-	-
bulnesol	1667	-	-	-	7.2	-	-
α-bisabolol *	1685	-	-	-	-	-	1.1
2,3-dihydrofarnesol	1695	-	-	-	2.4	-	-
2-pentadecanone *	1697	-	-	-	0.3	-	-
(*Z*,*Z*)-2,6-farnesol	1716	-	-	-	1.2	-	-
(*E*,*E*)-farnesol *	1720	-	-	-	2.6	-	-
(*E*,*Z*)-2,6-farnesol	1740	-	-	-	1.9	-	-
mint sulfide	1742	0.1	-	-	-	-	-
manoyl oxide	1993	-	-	1.6	5.0	-	-
*epi*-13-manoyl oxide	2010	-	-	-	0.8	-	-
abietatriene	2054	-	-	-	1.7	-	-
abietadiene	2081	-	-	-	8.3	-	-
Monoterpene hydrocarbons		75.7	6.5	59.6	46.3	4.0	2.4
Oxygenated monoterpenes		4.1	21.3	9.5	1.8	91.7	89.5
Sesquiterpene hydrocarbons		9.0	8.6	16.8	9.5	0.2	0.4
Oxygenated sesquiterpenes		10.0	53.0	9.5	21.4	0.5	2.1
Diterpene hydrocarbons		-	-	-	10.0	-	-
Oxygenated diterpenes		-	-	1.6	5.8	-	-
Phenylpropanoids		-	0.2	-	-	-	-
Sulfur derivatives		0.1	-	-	-	-	-
Other non-terpene derivatives		0.2	2.6	0.5	1.0	0.4	4.1
Total identified (%)		99.1	92.2	97.5	95.8	96.8	98.5

^a^ Linear retention indices on a HP-5MS capillary column; ^b^ Not detected; * Components for which the pure compound was injected for confirmation.

**Table 3:** In vitro antioxidant activities of the EOs of the six selected Algerian plants evaluated by 2,2′-diphenyl-1-picrylhydrazyl (DPPH), ferric-reducing/antioxidant power (FRAP) and 2,2′-azino-bis(3-ethylbenzothiazoline-6-sulphonic acid (ABTS) assays.

SAMPLE	DPPH (IC50; mg/mL)	FRAP (µmol TX/g sample)	ABTS (µmol TX/g sample)
*A. campestris*	7.80 ± 0.05 ^D^	2.48 ± 0.05 ^C^	7.01 ± 0.09 ^A^
*A. herba-alba*	2.61 ± 0.01 ^E^	8.17 ± 0.15 ^A^	6.74 ± 0.10 ^B^
*J. phoenicea*	15.15 ± 1.07 ^C^	2.85 ± 0.08 ^C^	5.50 ± 0.04 ^D^
*J. oxycedrus*	91.25 ± 3.40 ^A^	0.97 ± 0.03 ^D^	5.82 ± 0.15 ^C^
*M. pulegium*	3.07 ± 0.08 ^E^	5.31 ± 1.02 ^B^	6.67 ± 0.07 ^B^
*L. officinalis*	27.36 ± 1.25 ^B^	3.56 ± 0.09 ^C^	2.40 ± 0.01 ^E^
Ascorbic acid	0.0030 ± 0.0002	7101 ± 5.32	26,835.87 ± 11.245

Data (positive control excluded) were subjected to one-way ANOVA. Means within a column followed by different uppercase superscript letters are significantly different (*p* ≤ 0.05 according to Tukey′s test).

**Table 4:** Tyrosinase, acetyl and butyl cholinesterase (AChE and BuChE) inhibitory activities of the EOs of the six selected Algerian plants.

SAMPLE	Tyrosinase Inhibition Rate (%)	AChE Inhibition Rate (%)	BuChE Inhibition Rate (%)
*A. campestris*	38.36 ± 3.86 ^B,C^	53.95 ± 5.55 ^D^	14.27 ± 0.05 ^D^
*A. herba-alba*	31.35 ± 2.77 ^C^	56.60 ± 2.35 ^C,D^	72.03 ± 2.49 ^B^
*J. phoenicea*	11.35 ± 1.45 ^D^	40.57 ± 5.07 ^E^	6.47 ± 1.25 ^D^
*J. oxycedrus*	39.65 ± 3.72 ^B^	65.88 ± 2.15 ^B,C^	37.49 ± 3.95 ^C^
*M. pulegium*	30.76 ± 4.57 ^C^	67.69 ± 3.75 ^B^	95.53 ± 5.87 ^A^
*L. officinalis*	32.28 ± 1.01 ^B,C^	73.60 ± 3.85 ^A,B^	68.32 ± 4.25 ^B^
Kojic acid	87.54 ± 1.00 ^A^	-	-
Galantamine	-	82.40 ± 0.55 ^A^	97.1 ± 0.95 ^A^

The inhibition rates for all samples were calculated for their 1 mg/mL of concentration. The standard inhibitor kojic acid was only used at 0.05 mg/mL in the anti-tyrosinase assay. The standard inhibitor galantamine was also used at 0.004 mg/mL for anticholinesterase tests. BuChE: Butyrylcholinesterase; AChE: Acetylcholinesterase. Data were subjected to one-way ANOVA. Means within a column followed by different uppercase superscript letters are significantly different (*p* ≤ 0.05 according to Tukey′s test).

**Table 5:** Cytotoxic activities of the EOs of the six selected Algerian plants against two human breast adenocarcinoma (MCF-7 and T-47D) and one human colon cancer (Caco-2) cell lines. Results are expressed as LD_50_ (mg/mL) ± SD.

SAMPLE	MCF-7	T47D	Caco-2
*A. campestris*	0.28 ± 0.06 ^B^	0.43 ± 0.04 ^D^	0.76 ± 0.09 ^C^
*A. herba-alba*	0.016 ± 0.005 ^C^	0.08 ± 0.005 ^E^	0.19 ± 0.03 ^D^
*J. phoenicea*	0.32 ± 0.2 ^B^	0.64 ± 0.05 ^C^	0.98 ± 0.10 ^A,B^
*J. oxycedrus*	0.70 ± 0.02 ^A^	0.98 ± 0.04 ^A^	0.99 ± 0.07 ^A^
*M. pulegium*	0.37 ± 0.05 ^B^	0.64 ± 0.08 ^C^	0.91 ± 0.10 ^A,B,C^
*L. officinalis*	0.68 ± 0.04 ^A^	0.84 ± 0.07 ^B^	0.79 ± 0.03 ^B,C^
*Doxorubicin*	0.005 ± 0.0001 ^C^	0.009 ± 0.0004 ^E^	0.015 ± 0.003 ^D^

Data were subjected to one-way ANOVA. Means within a column followed by different uppercase superscript letters are significantly different (*p* ≤ 0.05 according to Tukey′s test).
